# Absence of Detectable Radionuclides in Breast Milk in Sendai, Japan in 2012 Even by High-Sensitivity Determination: Estimated Dose among Infants after the Fukushima Nuclear Disaster

**DOI:** 10.3390/ijerph18115825

**Published:** 2021-05-28

**Authors:** Zhaoqing Lyu, Sani Rachman Soleman, Tomoko Fujitani, Yukiko Fujii, Manal A. M. Mahmoud, Kouji H. Harada

**Affiliations:** 1 Department of Health and Environmental Sciences, Kyoto University Graduate School of Medicine, Kyoto 606-8501, Japan; lzq_1726362531@yahoo.co.jp (Z.L.); sani.soleman.67s@st.kyoto-u.ac.jp (S.R.S.); fujitani.tomoko.4w@kyoto-u.ac.jp (T.F.); 2 Department of Public Health, Faculty of Medicine, Universitas Islam Indonesia, Yogyakarta 55584, Indonesia; 3 Department of Pharmaceutical Sciences, Daiichi University of Pharmacy, Fukuoka 815-8511, Japan; yu-fujii@umin.ac.jp; 4 Department of Animal Hygiene and Environmental Sanitation, Faculty of Veterinary Medicine, Assiut University, Assiut 71526, Egypt; manalmahmoud@vet.au.edu.eg

**Keywords:** Fukushima Dai-ichi nuclear disaster, radionuclide, radiocesium, breastfeeding mother, infant exposure

## Abstract

The aim of this study was to estimate radionuclide levels in breast milk and the transferred dose to their infants in Sendai (100 km from Fukushima), Japan after the 2011 Fukushima nuclear disaster. Radionuclide concentrations were analyzed in 101 specimens of breast milk collected in 2012. Median values for minimum detectable activities were 0.39, 0.34, 1.1, 1.89, and 17.1 Bq/kg for ^137^Cs, ^134^Cs, ^131^I, ^110m^Ag, and ^40^K, respectively. Only radionuclides from ^40^K were detected. To estimate potential exposure and radiocesium dose, we assumed that the samples contained each minimum detectable activity level. The mean minimum detectable activity concentrations (standard deviation) of ^137^Cs and ^134^Cs were 0.42 (0.15) and 0.37 (0.14) Bq/kg, respectively. Means of estimated dietary intakes of ^137^Cs and ^134^Cs among infants were 0.35 (0.12) and 0.31 (0.11) Bq/day, respectively. The committed effective doses of radiocesium in infants aged 3 and 12 months via breastmilk were estimated at 5.6 (2.1) and 3.3 (1.2) μSv/year, respectively. Dietary intakes of ^137^Cs and ^134^Cs in breastfeeding mothers were back-calculated at 1.9 (0.71) and 1.7 (0.65) Bq/day, respectively. The study verified no discernible exposure to radionuclides among infants. The most conservative estimates were below the Japanese internal exposure limit of 1 mSv/year.

## 1. Introduction

Following a massive earthquake and tsunami on 11 March 2011 in northern Japan, the Fukushima Dai-ichi Nuclear Power Plant released radionuclides of rare gases, iodine, strontium, and cesium. The total amount of cesium-137 (^137^Cs) discharged into the environment was around 1.3 × 10^16^ Bq in the early stage, and was roughly 10% that of the Chernobyl disaster in 1986 [1,2]. Several areas within 50 km of the plant were restricted for residence because of the high radiation exposure estimated.

Radionuclides from radiocesium (^137^Cs and ^134^Cs) were dominant in the accident after the initial stage because of their longer half-lives (^134^Cs, 2.06 years and ^137^Cs, 30.1 years vs. ^131^I, 8 days). Soils and food crops were contaminated by radiocesium, resulting in exposure of residents in Fukushima and the neighboring areas. Dietary intake of radiocesium was assessed in the adult population [3,4,5] and measured just under 10 Bq/day, as confirmed using whole-body counters [6]. Lifetime cancer risks (either solid tumor or leukemia) from the radiation exposure among adults were estimated to be lower than cancer risks from lifestyle choices [5]. ^110m^Ag was a minor component of released radionuclides, but occasionally detected in contaminated soils [7]. ^40^K is a natural radionuclide contained in potassium and included in this study for comparisons with artificial radionuclides.

Few studies have been published on exposure to radionuclides via breastfeeding in the Fukushima accident. In April 2011, ^131^I was detected in breast milk of seven of 23 mothers residing within 250 km from the plant [8]. Infants were also exposed to cesium via breast milk; around 18% of absorbed cesium can transfer to milk at early lactation [9]. Because infants are more susceptible to the effects of radiation than adults, dietary exposure should be reduced as low as reasonably achievable. Whole-body measurements in infants and children in Fukushima showed no detectable radiocesium over 50 Bq/body after 2013, corresponding to 16 μSv/year for infants [10,11]. Actual dose levels among infants under the minimum detectable activity were still unclear. There was a symposium proceeding on screening analysis of breast milk of mothers in Fukushima [12], but the proceeding did not show any information on the minimum detectable activity (except for a description of detection limit at 2 Bq) and predicted internal doses. Detection limit of 2 Bq cannot provide more accurate estimation of internal dose than whole-body counting.

Dietary intake of radiocesium among adults was comparable in 2011 in areas located 50 and 100 km from the power plant in Miyagi and Fukushima [13], so we assumed that Sendai represents internal radiation exposure levels in areas 50–100 km from the power plant. Therefore, radionuclide concentrations found in breast milk in Sendai, 2012 were measured by high-sensitivity determination and the internal exposure among infants via breastfeeding was estimated. Thus, our study is the first sensitive analysis for radiocesium in breast milk samples in the Fukushima disaster.

## 2. Materials and Methods

### 2.1. Study Participants

Participants in this study were lactating mothers who gave birth at a general hospital in Sendai, Miyagi Prefecture between June and December of 2012 [14]. The hospital recorded 1229 deliveries in 2012 and 101 breast milk specimens were used to analyze levels of radionuclides. Written consent was obtained from all participants, who also responded to a questionnaire on demographic characteristics. Milk was collected within 2 months of delivery (mean: 326 g, range: 93–400 g). Specimens were archived in the Kyoto Human Specimen Bank [15]. The protocol for this study was approved by the Ethics Committee of Kyoto University Graduate School of Medicine and Faculty of Medicine and Hospital (approval number E25 on 1 February 2010, ‘Human exposure monitoring and risk assessment’).

### 2.2. Determination of Gamma Ray-Emitting Radionuclides

Cylindrical plastic containers were used to weigh and seal aliquots (range: 24–406 g) of breast milk. Radiometric determinations were performed using a high-purity, low-background, high-resolution germanium detector at the Radioisotope Research Center of Kyoto University (GMX-18200-S, EG&G ORTEC, CA, USA). The detection efficiency was 22.3% for 662 keV photon of ^137^Cs and energy resolution was 1.8 keV for 1.33 MeV photon of ^60^Co. The detector was covered by a lead shield (internal thickness: 10 cm) with 0.5 mm of electrolytic copper. A multichannel analyzer (4096 channels; range 0–3000 keV) was used to acquire the gamma spectrum (MCA8000; Princeton Gamma Tech Instruments, Princeton, NJ, USA). The characteristic gamma ray energies were monitored to identify and quantify radionuclides (^134^Cs, 604.7 and 795.9 keV; ^137^Cs, 661.7 keV; ^131^I, 364.5 keV; ^110m^Ag, 657.8 keV; ^40^K, 1461 keV). Gamma ray reference sources were used for calibration of the detector (QCY.44, Radiochemical Center, Buckinghamshire, UK). The samples were analyzed in within 30 days after collection with mean interval of 11 days, in 2012–2013. Average decays until measurement were 0.07%, 1%, 61% and 3% for ^137^Cs, ^134^Cs, ^131^I, and ^110m^Ag, respectively. Except for ^131^I, the decay before the analysis was negligible. To investigate the potential exposure to radiocesium in infants below the levels previously estimated by WBCs, the gamma spectrum of each sample was counted for >20,000 s. The Kaiser method with K = 3 was used to calculate the minimum detectable count rate [16], as shown in the following equation.

(1) $$n_{d}=\frac{k}{2}\left[\frac{k}{t_{s}}+\sqrt{{\left(\frac{k}{t_{s}}\right)}^{2}+4n_{b}\left(\frac{1}{t_{s}}+\frac{1}{t_{b}}\right)}\right]$$

where *n_d_* is net count rate of minimum detectable activity; *n_b_* is background count rate; *t_s_* is measurement time of sample; *t_b_* is measurement time of background. Background level was obtained in 300,000 s.

The activity of samples was calculated as shown in the following equation.

(2) $$A=\frac{n_{N}}{\varepsilon aW}\cdot f_{d}\cdot f_{sum}$$

where *n_N_* is net count rate of samples; *ε* is peak efficiency; *a* is emission ratio of characteristic gamma ray; *W* is sample mass; *f_d_* is physical decay correction factor; *f_sum_* is summing effect correction factor. *n_d_* was used instead of *n_N_* for samples with no detectable activity.

Measurement times of samples ranged from 23,620–100,740 s, with an average of 48,629 s. With an average sample mass of 324 g, measurement times of 5000, 20,000, 40,000 s gave minimal detectable activities of 0.82, 0.35 and 0.24 Bq/kg for ^137^Cs, respectively. Median values for the minimum detectable activity were 0.39, 0.34, 1.10, 1.89, and 17.1 Bq/kg for ^137^Cs, ^134^Cs, ^131^I, ^110m^Ag, and ^40^K, respectively. All activities were corrected to those on the sampling date. Procedural blanks were processed in parallel with each batch of 12 specimens to check for possible contamination.

### 2.3. Dose Estimation and Statistical Analysis

We assumed that infants were exclusively breastfed, no artificial infant formula was given, and specimens in which radionuclides were not detected were equal to the minimum detectable activity. Other exposure routes such as external radiation, house dusts, indoor and outdoor airs, drinking water and complementary food were not included. The daily intake of radionuclides in infants from 0 to 12 months was calculated from the mean human milk intake of 820 g/day, which had been reported for exclusively breastfed 3- to 4-month-old infants [17]. The mean milk intake at 12 months was also ca. 0.8 kg/day with larger variation than at 3- to 4-months [17]. Intake in breastfeeding mothers was estimated from a transfer rate to breast milk of 18% [9]. Radioactivity was converted into the effective dose using effective dose coefficients of ingestion for infants aged 3 months (0.026 μSv/Bq for ^134^Cs and 0.021 μSv/Bq for ^137^Cs), infants aged 1 year (0.016 μSv/Bq for ^134^Cs and 0.012 μSv/Bq for ^137^Cs), and mothers (0.019 μSv/Bq for ^134^Cs and 0.013 μSv/Bq for ^137^Cs) [18]. The estimation of annual dose was calculated assuming that the daily intake of radiocesium was constant over time. Effective dose coefficients were available only at 3 months and 1 year, and annual dose was calculated for each age.

Descriptive analyses of demographic characteristics, concentrations of radionuclides, and their doses were conducted. Data were summarized using JMP Pro 14 (SAS institute Inc., Cary, NC, USA).

## 3. Results and Discussion

Table 1 lists the characteristics of study participants. Mean maternal age was 31.5 years; this was comparable to the Japanese national average age at delivery (30.7 years at first delivery and 32.6 years at second delivery in 2016) [19]. More than one third (36%) were stay-at-home mothers (27% in 30–34 years at 2010 Japan census) and almost one fifth (17%) were medical practitioners (7.9% in 30–34 years at 2010 Japan census), perhaps indicating that their choice to participate in the study originated from a professional interest in radiation exposures. Smokers (current and ex-smokers) had 4.2 pack years. Alcohol consumption in ex-drinkers was 5.3 g ethanol/day.

We did not find detectable residues of radionuclides in any of the procedural blanks. Radionuclides were not detected (i.e., below the minimum detectable activity) in any milk specimens with the exception of ^40^K (detection rate: 30/101). ^131^I was not expected to be detected from breast milk samples, as it has a short half-life and was only detected at the early stage of the accident [8], and therefore focused on radiocesium and ^40^K to estimate radionuclide intake and dose. It is reasonable to suppose that the contribution of ^110m^Ag would be negligible, as the quantities released by the accident were smaller than those of ^137^Cs and as ^110m^Ag is not bioaccumulative in food products other than mollusks and arthropods [20]. Table 2 shows the distribution of radionuclide levels. Under the most conservative assumption that specimens without detectable levels of radionuclides contained the minimum detectable activity, the maximal levels of ^137^Cs and ^134^Cs were 1.7 and 1.6 Bq/kg, respectively. Potassium content in breast milk is approximately 0.5 g/kg [21] and natural potassium contains 30.4 Bq/g of ^40^K; we found similar ^40^K contents in the milk specimens. Variations in the minimum detectable activity mainly depended on sample mass (range: 24–406 g). Therefore, the assumption for the minimum detectable activity of each radionuclide might overestimate the real concentration.

Table 3 lists the estimates of dietary intake of radiocesium and committed effective dose in infants. Because radiocesium was not detected from all breast milk specimens, no potential exposure among infants could be considered. If the coefficients at 3 months and 1 year could be applicable to 0–6 months and 7–12 months, respectively, estimated mean dose would be an average of doses at both ages, which is 4.5 μSv/year. A previous study reported that the minimum detectable activity of radiocesium in infants aged 10–11 months was ≤2 Bq/kg of body weight by whole-body counting [10]. The calculated dose under conservative assumptions in that study was even lower than the previous estimate of the maximum (16 μSv/year for infants) [10]. In our study, the committed effective dose was far below the regulatory dose limit for general population in Japan of 1 mSv/year or 1000 μSv/year [22]. The dose from radiopotassium does not depend on intake. In natural potassium, the proportion of ^40^K is almost constant on Earth, and body content is homeostatic. The annual equivalent dose of ^40^K in the body is 165 µSv in adults and 185 µSv in children [23], which is much higher than radiocesium doses.

Dietary intake in mothers was back-calculated from the levels of radiocesium in breast milk specimens using a transfer ratio to milk of 18%. Table 4 lists the estimated intake and committed dose of radiocesium in mothers. Mean estimated intake of radiocesium was around 4 Bq/day, which is likely overestimated and still comparable to the intake reported in Fukushima near the power plant in 2011 and 2012 [3,4,5]. It plausibly explains why radiocesium was not detected in breast milk specimens at our sampling site, which is located approximately 100 km from the power plant. Compared with an annual ^40^K dose of 165 μSv/year in adults, the maximum annual dose of radiocesium was still low in this population (88 μSv/year).

In this study, high-sensitivity determination with large sample mass and long measurement times allowed sub-Bq/kg minimal detectable activity levels. The absence of detectable activity among the samples in this determination meant virtually no extra dose from breastfeeding related to the accident. Therefore, it is unlikely that residents of Sendai were internally exposed to notable radionuclides. Even if gestating women consumed products from the Fukushima prefecture, the estimated radiocesium levels in breast milk would be comparable to those we found and would not exceed the standard limit in Japan (1 mSv/year).

Although we used ca. 300 g of specimens and measured the radioactivity for >20,000 s using a high-resolution germanium detector, the actual levels of radiocesium could not be determined. More sensitive methods should be used in the future, such as extraction, concentration, and beta ray spectrometry. In addition to breast milk, placental transfer should be incorporated into exposure assessments for infants [24].

While it has been 10 years since the accident, the effects on the environment and society still persist. Since ^137^Cs and ^134^Cs have long half-lives that can affect human health for a number of years, it is still important to understand the exposure level to these radionuclides for radiological protection. The fact that an absence of detectable radionuclides has been observed in breast milk can deliver a better communication of risk to lactating mothers. After the accident, it was found that higher proportion of mothers had anxieties regarding radioactive contamination and turned to choosing formula feeding [25,26]. Even 4 years after the accident, the anxiety of mothers on radioactive contamination and breastfeeding continued [27,28]. Given the above, more information needs to be provided for lactating mothers. In this study, we also analyzed ^40^K in addition to artificial nuclides. Risk communication dictates that we inform the public of the existence of natural radiation [29].

This study analyzed breast milk samples collected in Sendai, Miyagi. Radiocesium deposition was higher in the Fukushima prefecture while dietary intake was comparable between Sendai and Fukushima [13]. In the initial period, residents in Fukushima were exposed to radioactive plumes. Although the internal burden of radionuclides in the initial period almost disappeared in 2012 [6], the difference in radiocesium deposition may affect the internal burden for this year. The result obtained in this study should be carefully interpreted to extrapolate it to Fukushima residents.

Health risks are not only from radiations, but also socio-economic changes after the disaster, which can affect the future health status of children such as those observed in adults for metabolic diseases [30,31]. Therefore, comprehensive studies on children’s health are warranted.

## 4. Conclusions

Artificial radionuclides could not be detected in any breast milk specimens from Sendai, excluding dietary exposure among infants. Most conservative estimates of radiocesium doses were low enough to be considered safe for infants in the sampling area.

## Figures and Tables

**Table 1:** Characteristics of study participants (*n* = 101).

Characteristics	Subcategory	*n* (%)/Mean (SD)
Age at participation (y)		31.5 (4.9)
BMI		22 (2.6)
Gestational week (wk)		38.9 (1.5)
Occupation (%)	Medical practitioners	17 (17)
	Service officers	25 (25)
	Stay-at-home mothers	36 (36)
	Clerical workers	19 (19)
	NA	4 (4)
Parity (%)	Nulliparous	58 (57)
	Multiparous 2	29 (29)
	Multiparous 3	8 (8)
	Multiparous 4	3 (3)
	NA	3 (3)
Delivery method (%)	Suction	14 (14)
	Cesarean	27 (27)
	Vaginal	56 (55)
	NA	4 (4)
Smoking (%)	Never	69 (68)
	Ex-smoker	28 (28)
	Current smoker	1 (1)
	NA	3 (3)
Drinking alcohol (%)	Never	29 (29)
	Ex-drinker	68 (67)
	Current drinker	0 (0)
	NA	4 (4)
Birth weight of infants (g)		3053 (479.2)
Lipid content of milk (%)		2.5 (1.1)

BMI, body mass index; NA, not available.

**Table 2:** Distribution of radionuclide levels (Bq/kg) in breast milk specimens (*n* = 101).

Nuclides	% of Detection	Mean	Median	SD	Min	Max
^137^Cs *	0	0.42	0.39	0.15	0.25	1.7
^134^Cs *	0	0.37	0.34	0.14	0.23	1.6
^40^K	29.7	19.6	18.2	7.1	13.1 **	73.5 **

* Radiocesium was not detected in any specimen, so the concentration was recorded as the minimum detectable activity. ** Radiopotassium was not detected, and the concentration was substituted with the minimum detectable activity.

**Table 3:** Estimated dietary intake of radiocesium via breastmilk and comitted effective dose in infants at 3 months and 1 year (*n* = 101).

	Dietary Intake (Bq/d)			Committed Effective Dose (μSv/y)	
	^134^Cs *	^137^Cs *	^40^K	^134^Cs + ^137^Cs (infants 3 mo **)	^134^Cs + ^137^Cs (infants 1 y **)
Mean(SD)	0.31(0.11)	0.35(0.12)	16.1(5.8)	5.6(2.08)	3.3(1.23)
Range	0.19–1.3	0.21–1.4	10.7–60.3	3.4–23.4	2–13.9
Median	0.28	0.32	15	5.1	3

* Radiocesium was not detected in any specimen. Minimum detectable activity was used for calculation. ** The committed effective dose from ingestion of radiocesium was calculated using effective dose coefficients of 0.026 μSv/Bq for ^134^Cs and 0.021 μSv/Bq for ^137^Cs in infants aged 3 months and 0.016 μSv/Bq for ^134^Cs and 0.012 μSv/Bq for ^137^Cs in infants aged 1 year. Daily intake of radiocesium was assumed to be constant over the course of the year.

**Table 4:** Estimated dietary intake of radiocesium and committed effective dose in breastfeeding women (*n* = 101).

	Dietary Intake (Bq/d) *		Committed Effective Dose for Breastfeeding Mothers (μSv/y) **
	^134^Cs	^137^Cs	^134^Cs + ^137^Cs
Mean(SD)	1.7(0.65)	1.9(0.71)	21.0(7.8)
Range	1.0–7.3	1.1–7.9	13–88
Median	1.5	1.8	19.2

* Dietary intake in breastfeeding mothers was back-calculated from the levels of radiocesium in breast milk specimens using a transfer ratio to milk of 18%. ** The committed effective dose from ingestion was calculated using effective dose coefficients of 0.019 μSv/Bq for ^134^Cs and 0.013 μSv/Bq for ^137^Cs in adults. Daily intake of radiocesium was assumed to be constant over the course of the year.

## Data Availability

The data presented in this study are available on request from the corresponding author. The data are not publicly available per the Ethical Guidelines for Medical and Health Research Involving Human Subjects in Japan.
